# Differential expression of RANK, RANK-L, and osteoprotegerin by synovial fluid neutrophils from patients with rheumatoid arthritis and by healthy human blood neutrophils

**DOI:** 10.1186/ar2137

**Published:** 2007-03-06

**Authors:** Patrice E Poubelle, Arpita Chakravarti, Maria J Fernandes, Karine Doiron, Andrée-Anne Marceau

**Affiliations:** 1 Centre de Recherche en Rhumatologie et Immunologie, Centre de Recherche du Centre Hospitalier de l'Université Laval (CRCHUL), 2705 boulevard Laurier, Ste-Foy, QC G1V 4G2, Canada

## Abstract

Functional links between bone remodeling and the immune system in chronic inflammatory arthritis are mediated, in part, by the ligand of receptor activator of nuclear factor-kappa-B (RANK-L). Because neutrophils play a crucial role in chronic inflammation, the goal of this study was to determine whether proteins of the RANK/RANK-L pathway are expressed by synovial fluid (SF) neutrophils from patients with rheumatoid arthritis (RA) and to characterize this pathway in normal human blood neutrophils. The expression of RANK-L, osteoprotegerin (OPG), RANK, and tumor necrosis factor receptor-associated factor 6 (TRAF6) was determined by polymerase chain reaction, enzyme-linked immunosorbent assay, Western blotting, and cytofluorometry. RANK signaling was analyzed by the degradation of inhibitor of kappaB-alpha (I-κB-α). SF neutrophils from patients with RA express and release OPG and express the membrane-associated forms of RANK-L and RANK. In contrast, normal blood neutrophils express only the membrane-associated form of RANK-L. They do not express the mRNAs encoding *OPG *and *RANK*. SF neutrophils from RA patients and normal blood neutrophils release no soluble RANK-L. They express the mRNA for *TRAF6*. The expression of OPG and RANK by normal human blood neutrophils, however, can be induced by interleukin-4 + tumor necrosis factor-alpha and by SFs from patients with RA. In contrast, SFs from patients with osteoarthritis do not induce the expression of OPG and RANK. Moreover, the addition of RANK-L to normal blood neutrophils pretreated by SF from patients with RA decreased I-κB-α, indicating that RANK signaling by neutrophils stimulated with SF is associated with nuclear factor-kappa-B activation. In summary, RANK-L is expressed by inflammatory and normal neutrophils, unlike OPG and RANK, which are expressed only by neutrophils exposed to an inflammatory environment. Taken together, these results suggest that neutrophils may contribute to bone remodeling at inflammatory sites where they are present in significantly large numbers.

## Introduction

Neutrophils, which are among the first cells to arrive in inflamed tissues, are activated during their margination and diapedesis across blood vessels and by cytokines at the site of inflammation [1]. They are involved in various chronic inflammatory diseases such as arthritis, active autoimmune colitis, and skin lesions of psoriasis [2,3]. In rheumatoid arthritis (RA), neutrophils are found in synovial fluids (SFs) and at the rheumatoid pannus-cartilage junction. They can degrade cartilage constituents [4,5]. The essential role of neutrophils in the initiation and maintenance of inflammation in the affected joints in RA was confirmed by the K/BxN mouse model of RA [6].

Besides their role in innate immunity, neutrophils act as antigen-presenting cells and regulate the adaptive immune response [7]. In the presence of certain cytokines, neutrophils acquire a variety of biological characteristics – such as the expression of major histocompatibility complex (MHC) class II antigens – that enable them to function as antigen-presenting cells [8,9]. In addition, phlogogenic cytokines activate neutrophils to express CCR6, CD80, CD83, CD86, and CD40, an expression pattern that resembles a dendritic-like phenotype [10,11]. The *in vitro *observation that neutrophils differentiate into dendritic-like cells has been corroborated *in vivo *by the demonstration that they express MHC class II, CD80, and CD86 proteins and that they can present antigens to T cells in an MHC class II-restricted manner in Wegener granulomatosis and RA [12,13]. The same inflammatory conditions that induce neutrophils to differentiate into dendritic-like cells have the capacity to delay the apoptosis of neutrophils, which are cells that are constitutively programmed for apoptotic cell death [14].

During the immune response, mature dendritic cells express receptor activator of nuclear factor-kappa-B (RANK) and tumor necrosis factor receptor-associated factor 6 (TRAF6) [15,16]. TRAF6 is an adapter protein implicated in signaling pathways of immunity and bone homeostasis [16]. RANK is activated by tumor necrosis factor-related activation-induced cytokine (TRANCE) [15]. TRANCE is a new member of the tumor necrosis factor (TNF) family and prevents apoptosis, increases survival, and stimulates cytokine production in dendritic cells [17,18]. TRANCE and the ligand of RANK (RANK-L) were originally cloned and sequenced from T lymphocytes [15,17]. They are also known as osteoprotegerin (OPG) ligand and osteoclast differentiation factor based on their capacity to induce osteoclastogenesis and activate osteoclasts via RANK [19-21]. Bone resorption is dependent upon osteoblast-osteoclast interactions that are mediated through the osteoblastic expression of a membrane form of RANK-L. This protein can also be processed into a soluble and active extracellular form [22]. In the presence of certain stimuli, cells can express both RANK-L and RANK, an observation reported in T lymphocytes [15,23]. These studies have shed light on the molecular and functional links between bone remodeling and the immune system. T lymphocytes, for instance, promote bone loss in inflammatory arthritis by expressing RANK-L that directly binds and activates osteoclasts [24].

The observation that neutrophils can differentiate into dendritic-like cells led us to test the hypothesis that inflammatory neutrophils could express proteins common to the local immune response and bone remodeling, such as those of the RANK/RANK-L pathway. To address this question, we investigated the expression of RANK-L, OPG, RANK, and TRAF6 mRNAs and proteins in neutrophils from the SF of patients with RA. Human blood neutrophils from healthy subjects were studied as normal control cells. Moreover, we demonstrate that the expression of genes of the RANK/RANK-L pathway could be induced by certain stimuli in neutrophils *in vitro*. The effect of SFs from patients with RA and from patients with osteoarthritis (OA) on the expression of these genes by normal neutrophils was also evaluated. Our observations suggest that the proteins of the RANK/RANK-L pathway expressed by neutrophils mediate important functions of neutrophils during the abnormal immune response and bone remodeling in RA.

## Materials and methods

### Reagents

Ficoll-Paque (1.077 density), RPMI 1640, Hanks' balanced salt solution (HBSS), and fetal bovine serum (FBS) were purchased from WISENT Inc. (St-Bruno, QC, Canada). Terminal deoxynucleotidyl transferase was purchased from Amersham Biosciences Inc. (now part of GE Healthcare, Little Chalfont, Buckinghamshire, UK). Trizol reagents and the Superscript™ II Reverse Transcriptase (RT) kit were obtained from Invitrogen Corporation (Carlsbad, CA, USA). Oligo-dT primers and *Taq *DNA polymerase were purchased from PerkinElmer Life and Analytical Sciences (Woodbridge, ON, Canada). The goat polyclonal anti-human RANK-L immunoglobulin (Ig) G antibody (Ab) (sc-7627) was purchased from Santa Cruz Biotechnology, Inc. (Santa Cruz, CA, USA). The mouse monoclonal anti-human RANK Ab was obtained from Alexis Biochemicals (part of Axxora Life Sciences, Inc., San Diego, CA, USA). The rabbit polyclonal anti-human inhibitor of kappaB-alpha Ab (no. 9242) was purchased from Cell Signaling Technology, Inc. (Danvers, MA, USA). Human recombinant RANK-L was obtained from PeproTech (Rocky Hill, NJ, USA). The human *RANK *cDNA was a kind gift from Dr. Naoki Sakurai (Discovery Research Laboratory, Tanabe Seiyaku Co., Ltd., Yodogawa-ku, Osaka, Japan).

### Cell preparation and culture conditions

The institutional review board of the Université Laval (Québec, QC, Canada) approved the present study, and volunteers signed a consent form. Samples were collected in anticoagulant solution, and cells were isolated under sterile conditions. Cells were obtained from the human venous blood of healthy donors and from the SF of seven patients with RA (according to the revised criteria of the American College of Rheumatology). Characteristics of patients with RA were as follows: six women/one man, age at onset of symptoms 52.1 ± 16.2 years (mean ± standard deviation [SD]), time between onset of symptoms and the present study 4.6 ± 4.0 years (mean ± SD), clinical parameters at the present examination: erythrocyte sedimentation rate (ESR) 27.6 ± 15.6 mm (mean ± SD), and C-reactive protein 36.2 ± 31.7 g/l (mean ± SD). Four patients were positive for IgM-rheumatoid factor (RF), and three were negative for IgM-RF. Four patients had radiographic erosions, two had local osteoporosis of inflammatory joints, and one showed no radiographic symptoms. Three patients had undergone no treatment, one was taking non-steroidal anti-inflammatory drugs, one was taking 5 mg/day prednisone, and two were taking disease-modifying anti-rheumatic drugs. Due to limited quantities of SF and neutrophils from patients with RA and due to the requirement of large numbers of cells depending on the experiments performed (described below), it was not possible to systematically include the cells of the seven patients in all the experiments reported.

Blood was centrifuged (250 *g*, 15 minutes) and the platelet-rich plasma was removed. The peripheral blood polymorpho (neutrophils) and mononuclear leukocyte (PBML) fractions were obtained by centrifugation over Ficoll-Paque after dextran sedimentation [25]. Remaining erythrocytes were eliminated by hypotonic lysis. SF neutrophils were directly obtained by centrifugation over Ficoll-Paque. After two washes, cells were counted and resuspended in culture medium. Differential cell counts of leukocytes were performed by cytofluorometry (EPICS-XL; Beckman Coulter, Fullerton, CA, USA) and Wright's and non-specific esterase stains. Neutrophil suspensions were more than 98% pure with no CD3-positive cells, and non-specific esterase-positive cells represented less than 0.2% of the cell population.

Cells were incubated in 12-well plates (2 ml/well) at 37°C and 5% CO_2 _for up to 4 days. Two culture media were studied. The control medium (CM) was RPMI 1640 and 10% FBS, and the survival medium (SM) consisted of CM supplemented with 500 pM granulocyte-macrophage colony-stimulating factor (GM-CSF), 10 ng/ml interleukin (IL)-4, and 10 ng/ml TNF-α. The cytokines present in SM were chosen for their anti-apoptotic effects on neutrophils [10,26,27]. Cells and supernatants were collected from days 1 to 4. After centrifugation (5,000 *g*, 2 minutes), cell pellets were resuspended in 1 ml of Trizol for RNA isolation or sonicated in 0.5 ml of HBSS (no. 211–512) for enzyme immunometric assay (EIA) analysis of cell-associated materials. These samples were frozen at -20°C until assayed. When required, samples of neutrophil supernatants were concentrated by centrifugation over Amicon Ultra 10000 MW CO (Millipore Corporation, Billerica, MA, USA) at 5,000 *g *for 1 hour at 4°C. Normal peripheral blood neutrophils (10^7^/ml) were also incubated at 37°C and 5% CO_2 _for 3 days in the presence of acellular SF from four of the seven RA patients described above and in the presence of acellular SF from two patients with OA. The incubation media consisted of 80% SF and 20% CM.

### Analysis by reverse transcriptase-polymerase chain reaction

Total RNA was isolated from cells by means of the Trizol reagent, and RT reaction was performed with Superscript™ II RT according to the manufacturer's instructions. The cDNAs were amplified by polymerase chain reaction (PCR) using gene-specific primer pairs designed with Primer 3 software (Whitehead Institute for Biomedical Research, Cambridge, MA, USA) (Table 1). Each PCR was performed with one tenth of the volume of cDNA from the RT reaction, 10 μM forward and reverse primers, 200 μM dNTPs, 2.5 μl 10× PCR buffer (200 mM Tris-HCl pH 8.4, 500 mM KCl), 1 to 1.5 mM MgCl_2_, 0.5 U *Taq *DNA polymerase, and autoclaved, distilled water to obtain a final volume of 25 μl. The number of cycles corresponding to the linear phase of amplification and the annealing temperature were optimized for each primer set (Table 1). The human β-actin transcript was used to standardize between PCRs. The PCR products were separated on a 1% agarose gel by electrophoresis in Tris acetic acid EDTA (ethylenediaminetetraacetic acid) buffer and visualized using ethidium bromide. The sequence of the amplified gene fragments was determined by direct sequencing.

**Table 1:** DNA sequences of the forward and reverse primers for the qualitative and semi-quantitative reverse transcriptase-polymerase chain reaction analyses

Gene identity	Accession number	Primer sequences^a^	Annealing temperature (°C)	Number of cycles (Quan.)^b^	Number of cycles (Qual.)^c^	Size of PCR product (bp)
*RANK-L*	AF019047	5'-CTG-ATG-AAA-GGA-GGA-AGC-AC-3'	65	29	35	546
		5'-GAT-GAC-ACC-CTC-TCC-ACT-TC-3'				
*OPG*	U94332	5'-TGC-TGT-TCC-TAC-AAA-GTT-TAC-G-3'	56	35	40	433
		5'-CTT-TGA-GTG-CTT-TAG-TGC-GTG-3'				
*RANK*	AF018253	5'-CCT-GGA-CCA-ACT-GTA-CCT-TC-3'	58	34	40	500
		5'-TTC-CTC-TAT-CTC-GGT-CTT-GC-3'				
*TRAF6*	U78798	5'-TGA-TAG-TGT-GGG-TGG-AAC-TG-3'	58	27	35	456
		5'-CTC-CTT-GGA-CAA-TCC-TTC-AG-3'				
*β-Actin*	NM001101	5'-CGT-GAC-ATT-AAG-GAG-AAG-CTG-TGC-3'	58	21	28	375
		5'-CTC-AGG-AGG-AGC-AAT-GAT-CTT-GAT-3'				

^a^For each pair of sequences, the forward primer appears first and the reverse primer appears second. ^b^The number of cycles used in the semi-quantitative (quan.) PCR experiments corresponds to the linear phase of the amplification reaction. ^c^The number of cycles used in the qualitative (qual.) PCR experiments was increased to allow the possible detection of the mRNA. OPG, osteoprotegerin; PCR, polymerase chain reaction; RANK, receptor activator of nuclear factor-kappa-B; RANK-L, ligand of receptor activator of nuclear factor-kappa-B; TRAF6, tumor necrosis factor receptor-associated factor 6.

### EIA analysis of RANK-L and OPG

The EIAs used were at two sites with horseradish peroxidase (HRP) as a tracer. Ninety-six-well plates were coated with either the human OPG/Fc Chimera (805-OS; R&D Systems, Inc., Minneapolis, MN, USA) or a monoclonal anti-human OPG Ab (MAB8051; R&D Systems, Inc.) in phosphate-buffered solution (pH 7.4). A biotinylated secondary goat anti-human RANK-L Ab (BAF626; R&D Systems, Inc.) or a compatible biotinylated secondary goat anti-human OPG Ab (BAF805; R&D Systems, Inc.) in phosphate-buffered solution (pH 7.4) containing bovine serum albumin (BSA) was used. Antigen-Ab complexes were detected by the addition of a streptavidin-HRP conjugate and tetramethylbenzidine as a substrate for HRP. Concentrations of RANK-L and OPG were obtained from a standard curve generated by known concentrations of human RANK-L and OPG. The detection limits were 15 and 7.5 pg/ml for RANK-L and OPG, respectively.

### Western blot analysis

SF neutrophils (3 × 10^6^) from four patients with RA were solubilized in SDS sample buffer. The positive control was COS-7 cells transiently transfected (Fugene 6 transfection reagent; Roche Diagnostics, Indianapolis, IN, USA) with human *RANK *cDNA. Transfected cells were lysed in 1.5% Triton X-100 at 4°C for 5 minutes, and samples were analyzed on a 7% SDS-polyacrylamide gel. The proteins were transferred to a polyvinylidene difluoride (PVDF) membrane (Millipore Corporation) at 4°C overnight. The membrane was blocked with 2% pig gelatin in tris-buffered saline (TBS) with 6% Tween 20 for 60 minutes, incubated with 0.2 μg/ml of a goat anti-human RANK IgG Ab (no. AF683; R&D Systems, Inc.), washed three times in TBS-Tween, and incubated with 0.04 μg/ml of a rabbit HRP-conjugated anti-goat IgG Ab (The Jackson Laboratory, Bar Harbor, ME, USA). After incubation in SF from patients with RA for 3 days (see above), healthy blood neutrophils were centrifuged at 600 *g *for 30 minutes on a percoll gradient to remove debris and dead cells [28], washed, resuspended in HBSS (15 × 10^6 ^cells per milliliter), and stimulated at 37°C by 50 ng/ml TNF-α for 10 minutes or by 100 ng/ml RANK-L for 10 and 20 minutes. They were then transferred to 2× boiling Laemmli's sample buffer (1×: 62.5 mM Tris/HCl [pH6.8], 4% [wt/vol] SDS, 5% [vol/vol] 2-mercaptoethanol, 8.5% [vol/vol] glycerol, 2.5 mM orthovanadate, 10 mM para-nitrophenylphosphate, 10 μg/ml leupeptin, 10 μg/ml aprotinin, and 0.025% bromophenol blue). Proteins were separated on a 12% SDS-PAGE gel and transferred on a PVDF membrane. Immunoblotting was performed using 5% Blotto as a blocking agent. The primary Ab directed against I-κB-α was diluted 1:1,000 in TBS-Tween 5% BSA and incubated with the membrane for 1 hour. The goat HRP-conjugated anti-rabbit IgG Ab (The Jackson Laboratory) was diluted 1:20,000 and incubated with the membrane. The labeled Abs were detected by the ECL (enhanced chemiluminescence) detection system (GE Healthcare) and visualized on Kodak Biomax MR film (Eastman Kodak, Rochester, NY, USA).

### Cytofluorometry

The expression of RANK-L and RANK at the membrane was evaluated by cytofluorometry. Freshly separated healthy human blood or SF neutrophils from patients with RA and healthy neutrophils incubated in SFs were incubated with a goat anti-human RANK-L Ab (Santa Cruz Biotechnology, Inc.) followed by a fluorescein isothiocyanate (FITC)-conjugated anti-goat F(ab')_2 _Ab. A normal goat IgG was used as control. To evaluate cell surface expression of RANK, healthy human blood neutrophils incubated in SFs were fixed and permeabilized using the Fixation/Permeabilization Solution kit (no. 554723) from BD Biosciences Pharmingen (San Diego, CA, USA). Briefly, non-specific staining of Fc receptors was blocked by 10% human decomplemented serum before cells were resuspended in 250 μl of Fixation/Permeabilization Solution (BD Cytofix/Cytoperm no. 554714) for 45 minutes at 4°C. Cells were then washed and permeabilized with BD Perm/Wash buffer in the presence of 10% mouse non-specific immune serum. Fixed and permeabilized neutrophils were then stained with a mouse monoclonal anti-human RANK Ab (ALX-804-212-C100) followed by a FITC-conjugated anti-mouse F(ab')_2 _Ab. Corresponding controls with non-specific Abs were also performed.

### Viability

Neutrophil viability was evaluated by the lactate dehydrogenase (LDH) release assay. Neutrophil suspensions after incubation were centrifuged (5,000 *g*, 1 minute). Supernatants and pelleted neutrophils were collected separately, and cells were lysed in 1% Triton X-100 buffer. Prior to colorimetric analysis at 340 nm, 1.25 ml of substrate (0.14 mg/ml NADH in 0.1 M sodium phosphate buffer, pH 7.35) and 50 μl of pyruvate solution were added to 50 μl of cell lysate or supernatant. Results were expressed as percentages of the ratio between the optical density values measured in supernatants and the total optical density value measured in cells plus supernatants. Viable neutrophils that did not release LDH at day 3 represented 32% and 39% in CM and SM, respectively.

### Statistics

Values were expressed as means ± standard error of the mean (SEM) of *n *experiments performed with cells from different donors. Statistical analyses were performed using GraphPad Instat 3.0 (GraphPad Software, Inc., San Diego, CA, USA). Non-parametric analysis with the Mann-Whitney test was used to compare the means of two groups. Paired groups were analyzed using the paired *t *test. Significance was set at a two-tailed *p *value of less than 0.05.

## Results

### Expression of *RANK-L*, *OPG*, *RANK*, and *TRAF6 *mRNAs by SF neutrophils from patients with RA and by healthy human blood cells

**Figure 1:**
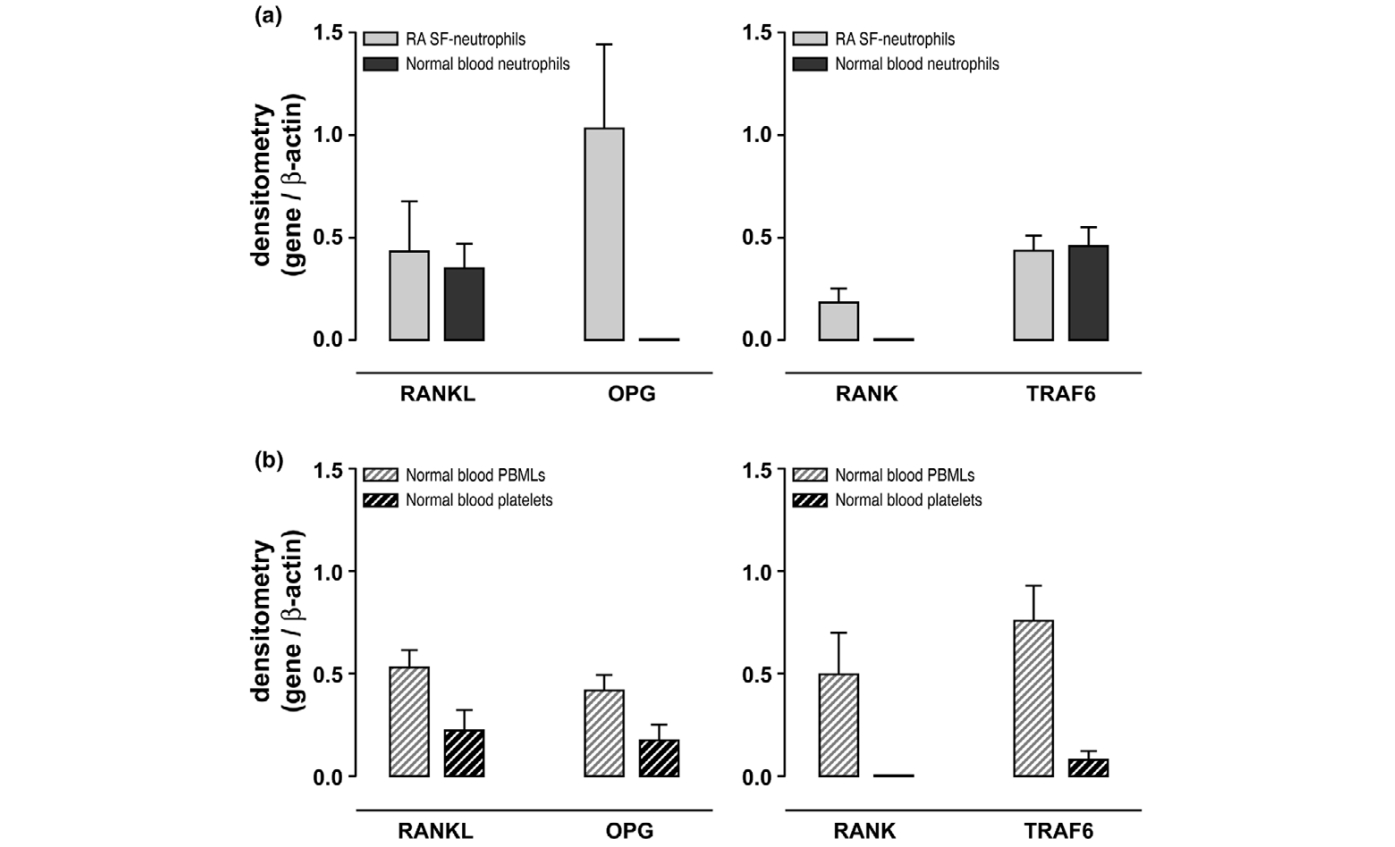
Expression of *RANK-L*, *OPG*, *RANK*, and *TRAF6 *mRNAs by synovial fluid (SF) neutrophils isolated from patients with rheumatoid arthritis (RA) and by normal blood cells. **(a) **Purified neutrophils of SF from four patients with RA and of blood from seven normal donors were evaluated by semi-quantitative reverse transcriptase-polymerase chain reaction (RT-PCR) analyses. **(b) **Purified peripheral blood mononuclear leukocytes (PBMLs) and platelets of blood from seven normal donors were evaluated by semi-quantitative RT-PCR analyses. To avoid leukocyte contamination in the platelet suspension, platelets were isolated from the upper part of the platelet-rich plasma (PRP). After centrifugation (600 *g*, 30 minutes), the pellets were resuspended in Trizol. No contaminating leukocytes in the upper part of the PRP were observed under light microscopy after Wright's stain. Histograms represent mean ± standard error of the mean of ratios of densitometric values for RANK-L/β-actin, OPG/β-actin, RANK/β-actin, and TRAF6/β-actin. OPG, osteoprotegerin; RANK, receptor activator of nuclear factor-kappa-B; RANK-L, ligand of receptor activator of nuclear factor-kappa-B; TRAF6, tumor necrosis factor receptor-associated factor 6.

Freshly isolated neutrophils from SF of patients with RA expressed RANKL, OPG, TRAF6, and RANK as determined by semi-quantitative RT-PCR (Figure 1a). In contrast, freshly isolated peripheral blood neutrophils from healthy subjects expressed RANK-L and TRAF6, but not OPG and RANK (Figure 1a). PBMLs from healthy subjects expressed the four genes tested, and platelets expressed RANK-L, TRAF6, OPG, and not RANK (Figure 1b). The absence of OPG or RANK expression in healthy neutrophils was confirmed by qualitative PCR using an increasing number of cycles as indicated in Table 1 (data not shown). The fact that PBMLs expressed *OPG *and *RANK *mRNA enabled us to confirm that the neutrophil and platelet preparations were not contaminated by these cells.

### Expression of RANK-L, OPG, and RANK proteins by SF neutrophils from patients with RA

**Figure 2:**
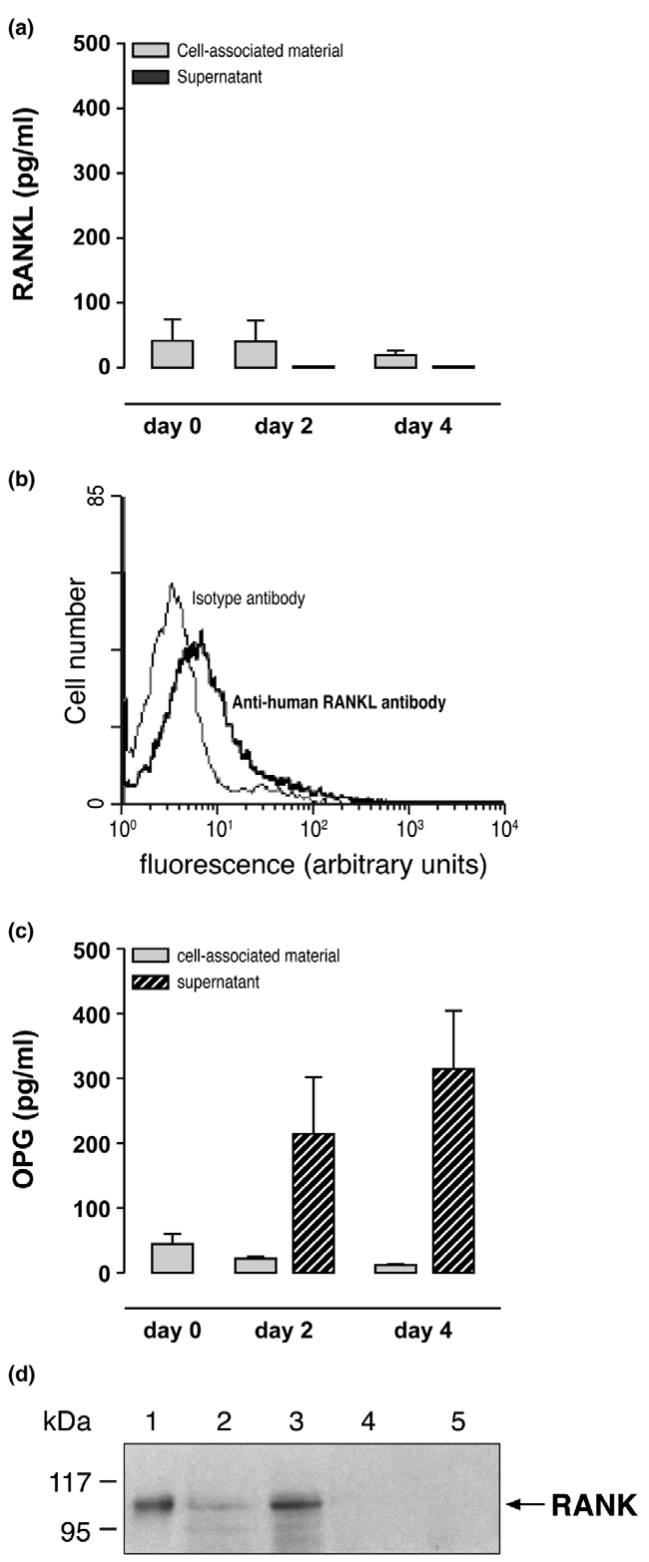
Expression of RANK-L, OPG, and RANK proteins by synovial fluid (SF) neutrophils from patients with rheumatoid arthritis (RA). **(a) **Cell-associated materials and supernatants of SF neutrophils (10^7^/ml) from three patients with RA were analyzed by enzyme immunometric assays (EIAs) for RANK-L at day 0 and after 2 and 4 days of incubation in control medium. **(b) **Surface expression of RANK-L by freshly isolated SF neutrophils from patients with RA. Flow cytometry was performed after incubation of neutrophils with a goat anti-human RANK-L antibody followed by a fluorescein isothiocyanate-conjugated anti-goat F(ab')_2 _antibody. Control isotype antibody was a normal goat immunoglobulin G (IgG). Results shown are representative of SF neutrophils from three patients with RA. **(c) **Samples similar to those in **(a) **were analyzed by EIAs for OPG. **(d) **Cell-associated materials of SF neutrophils from four patients with RA were solubilized in SDS sample buffer and subjected to SDS-PAGE under reducing conditions (lanes 2 to 5). Protein loading values in lanes 2, 3, 4, and 5 were 123, 163, 135, and 125 μg, respectively. COS-7 cells transfected with a human *RANK *cDNA were used as a positive control (lane 1). Western blotting was performed with a goat anti-human RANK antibody, a horseradish peroxidase-conjugated anti-goat IgG antibody, and the enhanced chemiluminescence detection system. The position of the molecular weight markers in kilodaltons is indicated on the left. OPG, osteoprotegerin; RANK, receptor activator of nuclear factor-kappa-B; RANK-L, ligand of receptor activator of nuclear factor-kappa-B.

Freshly isolated neutrophils from SF of patients with RA expressed not only the mRNA of the four genes studied (Figure 1a) but also the corresponding proteins RANK-L, OPG, and RANK (Figure 2). Cell-associated materials of SF neutrophils from patients with RA contained detectable amounts of RANK-L and OPG as measured by EIAs (Figure 2a,c). In contrast, cell-associated materials of healthy blood neutrophils contained 68 ± 13 pg/ml RANK-L and no OPG (*n *= 13). SF neutrophils obtained from patients with RA and incubated for up to 4 days in CM (as described in Materials and methods and Figure 2a) or in SM (data not shown) did not release RANK-L. However, freshly isolated SF neutrophils of patients with RA, as well as healthy blood neutrophils, expressed RANK-L protein on their plasma membrane, as evaluated by cytofluorometry (Figure 2b). Percentages of RANK-L-positive neutrophils freshly isolated from SF of patients with RA (*n *= 5) and from normal blood (*n *= 13) were 10.9% ± 4.3% and 2.9% ± 0.7%, respectively (*p *= 0.09). Moreover, the same SF neutrophils obtained from patients with RA and incubated for up to 4 days in CM showed a time-dependent release and accumulation of OPG in supernatants (Figure 2c). Finally, the expression of RANK protein was also determined by Western blot analysis in freshly isolated SF neutrophils of four patients with RA. The SF neutrophils of two of the four patients studied expressed a detectable band of an apparent molecular weight of 110 kDa. This band is specific for the polyclonal anti-human RANK Ab as shown in COS cells transiently transfected with a human *RANK *cDNA (Figure 2d). The amounts of OPG and RANK-L in SFs of the same patients with RA were also quantitated by EIA. SFs of patients with RA contained 6,990 ± 1,912 pg/ml OPG and 21 ± 12 pg/ml RANK-L (mean ± SEM, *n *= 7) with a RANK-L/OPG ratio of 0.003.

### Normal human blood neutrophils can acquire the capacity to express OPG and RANK

**Figure 3:**
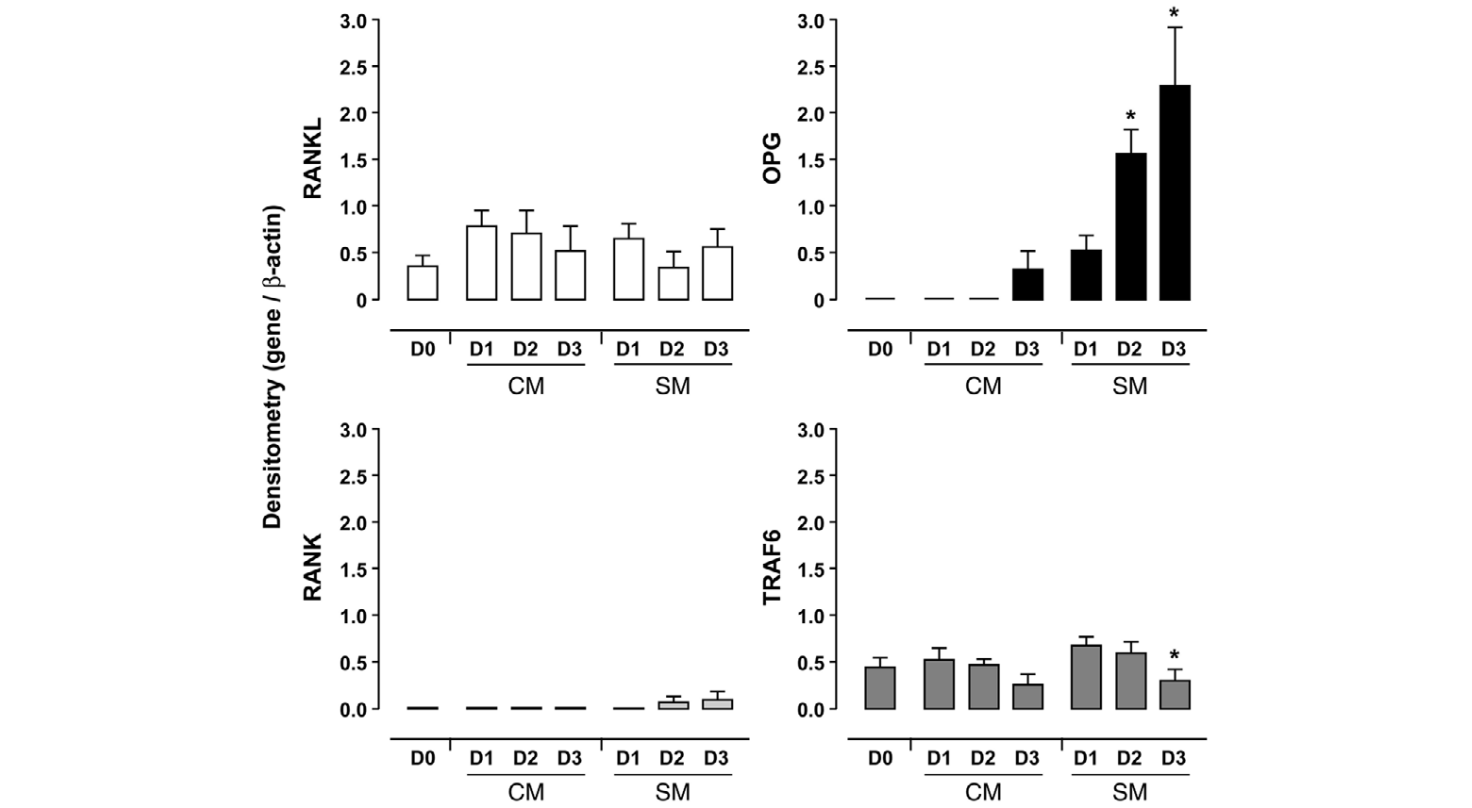
Effect of cytokines on the expression of *RANK-L*, *OPG*, *RANK*, and *TRAF6 *mRNAs by normal human blood neutrophils *in vitro*. Purified neutrophils were incubated in control medium (CM) or in survival medium (SM) for 1 to 3 days (D1, D2, D3). After RNA extraction, semi-quantitative reverse transcriptase-polymerase chain reaction analysis was performed. Histograms represent mean ± standard error of the mean of ratios of densitometric values for RANK-L/β-actin, OPG/β-actin, RANK/β-actin, and TRAF6/β-actin (*n *= 5 normal donors). Student's paired *t *test: *p < 0.05 (D3 versus D1, D2 versus D1). OPG, osteoprotegerin; RANK, receptor activator of nuclear factor-kappa-B; RANK-L, ligand of receptor activator of nuclear factor-kappa-B; TRAF6, tumor necrosis factor receptor-associated factor 6.

We next investigated whether *in vitro *conditions could mimic our *in vivo *observations (Figures 1 and 2). Neutrophils were incubated with cytokines that decrease neutrophil apoptosis and that are found in SFs from patients with RA [29]. Healthy blood neutrophils were shown to express *RANK-L *mRNA under CM and SM conditions without any significant changes from day 1 to day 3 (Figure 3). The expression of *OPG *mRNA by neutrophils incubated in CM was not yet detectable on day 2 and appeared only after 3 days (Figure 3). The incubation of neutrophils in SM, however, strikingly upregulated the expression of this gene. The expression of *OPG *mRNA, which was absent at day 0 (Figure 1a), significantly increased at days 1, 2, and 3 (Figure 3). Control studies using different combinations of the cytokines were also conducted. Neutrophils incubated for 3 days in medium containing TNF-α alone expressed RANK-L but not OPG. When IL-4 was present, alone or in combination with GM-CSF or TNF-α, neutrophils expressed OPG with no change of RANK-L. GM-CSF alone had no effect on the expression of the genes tested (data not shown). Healthy blood neutrophils that do not express RANK at day 0 (Figure 1a) have the capacity to express *RANK *mRNA *in vitro *when incubated in SM (Figure 3). The expression of RANK by neutrophils was detectable from day 2 to day 3 (results observed in two donors out of nine healthy subjects studied). The expression of TRAF6 by normal blood neutrophils, on the other hand, was detected in all the conditions tested but decreased significantly at day 3 in the presence of SM (Figure 3). In contrast, PBMLs expressed OPG and RANK from day 0 (Figure 1b) to day 3 (data not shown). In these cells, a decrease in the expression of OPG and RANK was observed when incubated in CM and an increase was observed when incubated in SM. TRAF6 expression by PBMLs was similar in CM and in SM with no significant changes from days 1 to 3 (data not shown).

These findings were then confirmed at the protein level. Incubation of healthy blood neutrophils in CM or SM conditions for up to 3 days did not modify the membrane expression of RANK-L as evaluated by cytofluorometry and did not stimulate the release of detectable amounts of OPG in neutrophil supernatants as measured by EIA (data not shown). Prolongation of the incubation time up to 5 days in SM, however, led to an accumulation of OPG in the supernatants of these neutrophils (2.0 ± 0.4 pg/ml, *n *= 7). Moreover, the same neutrophil supernatants contained no RANK-L as evaluated by EIA (data not shown). In contrast, PBMLs cultured under similar conditions secreted RANK-L and OPG in the supernatants (data not shown), confirming that neutrophils and PBMLs expressed RANK-L and OPG differently.

### SF from patients with RA activates the expression of RANK-L, OPG, and RANK in normal blood neutrophils

**Figure 4:**
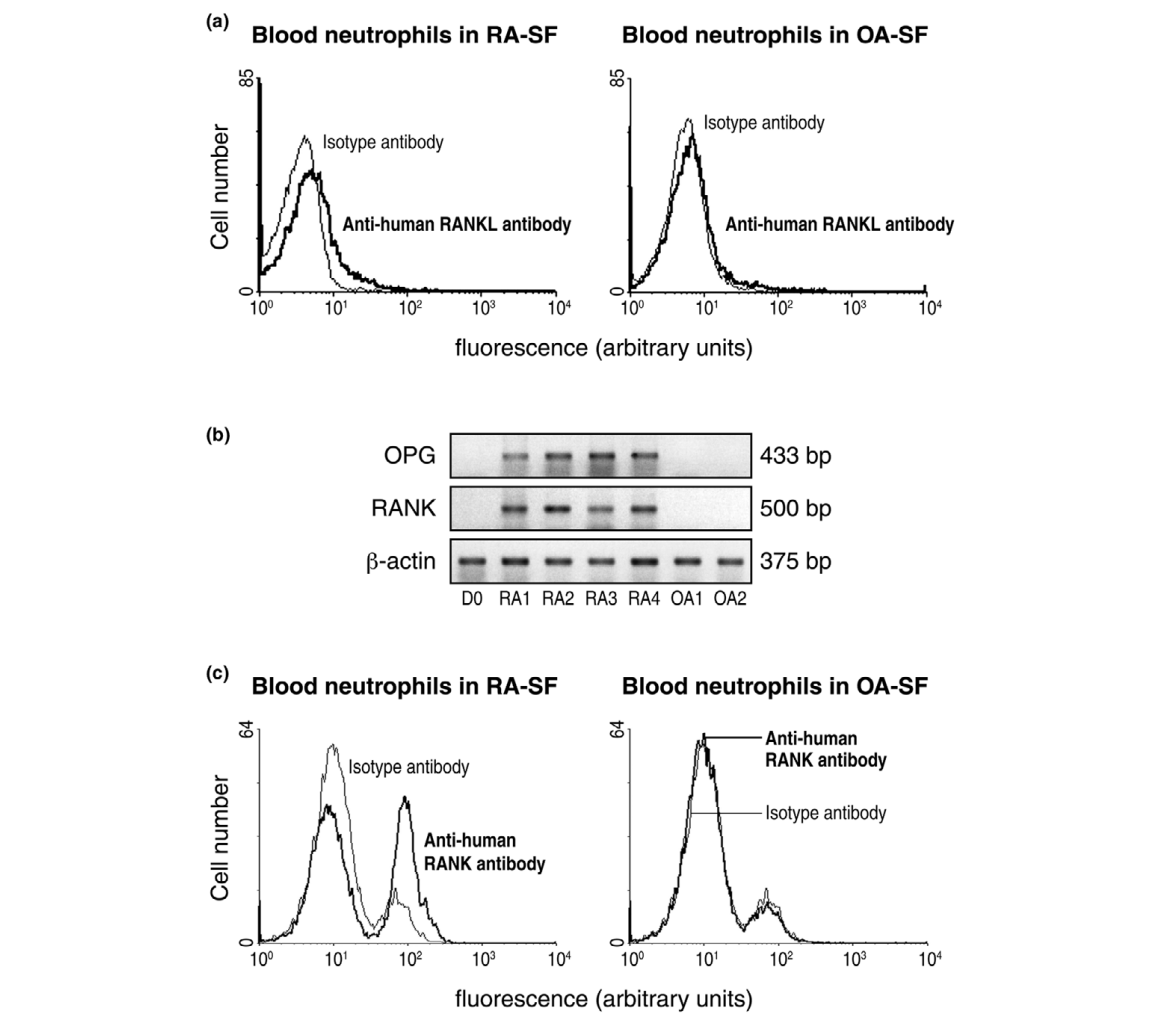
Induction of the expression of RANK-L, OPG, and RANK by normal human blood neutrophils incubated in the presence of synovial fluid (SF) from patients with rheumatoid arthritis (RA) or patients with osteoarthritis (OA). **(a) **Surface expression of RANK-L by normal blood neutrophils incubated in SF from patients with RA (RA-SF) or OA (OA-SF) for 2 days. Flow cytometry was performed after incubation of neutrophils with a goat anti-human RANK-L antibody followed by a fluorescein isothiocyanate (FITC)-conjugated anti-goat F(ab')_2 _antibody. Control isotype antibody was a normal goat immunoglobulin G (IgG). Results shown are representative of three RA-SF and nine OA-SF. **(b) **Expression of mRNA for *OPG *and *RANK *by normal blood neutrophils incubated for 2 days in SF from four patients with RA and two patients with OA. Total RNA was isolated from freshly isolated normal blood neutrophils (D0) and from neutrophils of the same healthy donors after 2 days of incubation in SF (RA-1 to -4, OA-1, -2). RNA was then analyzed by reverse transcriptase-polymerase chain reaction. Results shown are representative of two different healthy donors. **(c) **Surface expression of RANK by normal blood neutrophils incubated in SF from patients with RA (RA-SF) or OA (OA-SF) for 3 days. Flow cytometry was performed after cellular fixation, permeabilization, and staining with a mouse monoclonal anti-human RANK antibody followed by a FITC-conjugated anti-mouse F(ab')_2 _antibody. Control isotype antibody was a non-specific mouse IgG. Results shown are representative of neutrophils from two different healthy subjects incubated in three different RA-SF and two OA-SF. OPG, osteoprotegerin; RANK, receptor activator of nuclear factor-kappa-B; RANK-L, ligand of receptor activator of nuclear factor-kappa-B.

Having established that inflammatory cytokines can stimulate healthy blood neutrophils to express OPG and RANK (Figure 3) and that SF neutrophils from patients with RA spontaneously expressed RANK-L, OPG, and RANK proteins (Figure 2), we next investigated the effect of SF on the expression of these genes by incubating healthy human blood neutrophils in the presence of SF from patients with RA (Figure 4). After 2 days of incubation of healthy blood neutrophils in medium containing 80% SF from patients with RA and 20% CM, membrane RANK-L significantly increased and was detected on 13.4% ± 4.7% of cells (*n *= 5) (versus 2.5% ± 0.8% neutrophils in CM alone, a percentage similar to that of freshly isolated neutrophils). Similar experiments with incubation medium containing SF from patients with OA revealed that 3.9% ± 0.5% of neutrophils expressed membrane RANK-L (*n *= 14) (Figure 4a). The difference in the expression of membrane RANK-L in healthy blood neutrophils incubated in SF from patients with RA versus patients with OA was significant (*p *= 0.019). Moreover, SF from patients with RA, but not patients with OA, activated healthy blood neutrophils to express *OPG *and *RANK *mRNAs as evaluated by RT-PCR (Figure 4b). Finally, SFs from patients with RA, but not from patients with OA, strongly activated healthy blood neutrophils to express RANK at the cell surface. Membrane RANK, which is not expressed by freshly isolated human blood neutrophils (data not shown), was detected on 15.3% ± 5.6% of cells after 3 days of incubation in the presence of SF of patients with RA (Figure 4c).

**Figure 5:**
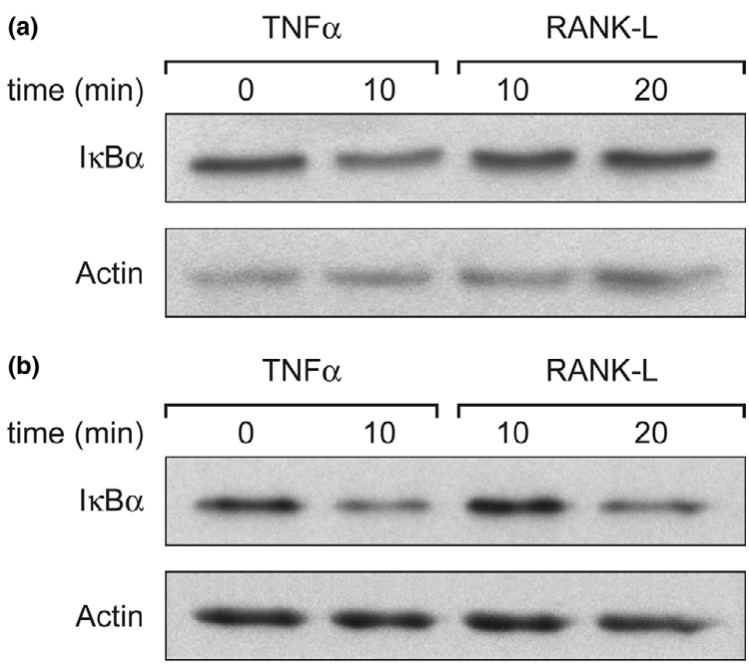
Degradation of inhibitor of kappaB-alpha (I-κB-α) in RANK-L-activated neutrophils. I-κB-α was detected in whole-cell lysates by Western blotting as described in Materials and methods. Freshly isolated neutrophils **(a)**, or blood neutrophils pretreated for a 3-day incubation period in rheumatoid arthritis-synovial fluid (RA-SF) (80%) + control medium (20%) **(b)**, were stimulated with 50 ng/ml tumor necrosis factor-alpha (TNF-α) for 10 minutes or with 100 ng/ml RANK-L for 10 and 20 minutes. Western blotting was performed with a rabbit anti-human I-κB-α antibody, a horseradish peroxidase-conjugated anti-rabbit immunoglobulin G antibody, and the enhanced chemiluminescence detection system. Results shown are representative of neutrophils from three different healthy subjects incubated in three different RA-SF. RANK-L, ligand of receptor activator of nuclear factor-kappa-B.

The release of active nuclear factor-kappa-B (NF-κB) secondary to the stimulation of RANK by RANK-L is associated with the phosphorylation of the inhibitory I-κB-α protein. Subsequently, I-κB-α decreased through its conjugation with ubiquitin and its degradation by proteasome. To determine whether RANK expressed on the surface of neutrophils was functional, healthy blood neutrophils preincubated 3 days in SF from patients with RA were stimulated by RANK-L (or TNF-α as a positive control) and total amounts of I-κB-α protein were evaluated by Western blotting. A time-dependent decrease of I-κB-α protein was demonstrated in the presence of RANK-L or TNF-α (Figure 5), confirming that the stimulation of cell surface RANK in neutrophils pretreated with SF from patients with RA was followed by intracellular signaling through, at least in part, the NF-κB pathway.

## Discussion

The present report is the first to demonstrate that neutrophils have the capacity to express proteins of the RANK pathway. We observed the expression of the membrane-associated form of RANK-L in healthy blood neutrophils. In contrast, SF neutrophils from patients with RA not only express the membrane-associated form of RANK-L but also express RANK and secrete OPG. Remarkably, healthy human blood neutrophils can be induced to express RANK and OPG in response to different stimuli such as IL-4+TNF-α and SF from patients with RA. The RANK protein expressed on the surface of neutrophils stimulated by SF from patients with RA is functional since it can be activated in the presence of RANK-L. Interestingly, TRAF6 is expressed by both inflammatory and healthy neutrophils and its expression is not modulated by any stimulus. These findings may have important pathophysiological implications considering that neutrophils are present in large numbers at inflammatory sites and are involved in cell-cell interactions in inflamed tissues.

The fact that SF and blood neutrophils express RANK-L as membrane materials and that neutrophils incubated *in vitro *for up to 4 days generated no soluble RANK-L (Figure 2a) allow us to consider neutrophils as a new cell type that generates RANK-L without any release in the extracellular milieu. From that point of view, neutrophils are different from other cell types such as osteoblasts, fibroblasts, or T lymphocytes, which produce RANK-L and release soluble RANK-L after stimulation [17,24,30,31]. In the context of a chronic inflammatory reaction, RANK-L/RANK interactions between T lymphocytes and dendritic cells and between T lymphocytes and osteoclasts explain the role of T cells in disease progression [17,24,32]. The enhancing effect of SF from patients with RA on the expression of neutrophil membrane RANK-L (Figure 4a) should not be neglected in terms of cell-cell interactions. Neutrophils have been described at sites of rheumatoid pannus invasion into cartilage and subchondral bone [5]. Thus, infiltrating neutrophils that, therefore, are numerous and implicated in the local inflammatory process of active immune diseases could also directly impact on the local immune and bone remodeling responses through their membrane RANK-L. The rheumatoid pannus-bone junction at sites of subchondral bone destruction showed local RANK-L expression that was more prevalent in active RA [33]. The cellular sources of RANK-L in these rheumatoid bone destruction sites were not all identified with no mention of neutrophils [33]. The present data on the increased RANK-L expression by RA neutrophils, together with the presence of neutrophils at the pannus-bone interface [34], suggest that through cell-cell interactions such inflammatory neutrophils could activate RANK-expressing osteoclasts and bone resorption.

The capacity of neutrophils freshly isolated from inflammatory SFs to express large quantities of OPG (Figures 1a and 2c) in comparison to the inferior amount of OPG expressed by healthy blood neutrophils after incubation with certain stimuli (Figure 3) suggests that the induction of OPG expression by neutrophils is regulated by multiple factors. *In vitro*, the maximal concentration of OPG released by neutrophils in the presence of IL-4, TNF-α, and GM-CSF was approximately 2 pg/ml. In contrast, OPG concentrations spontaneously released in supernatants of SF neutrophils were 200 to 300 pg/ml. It follows that, if the cytokine combination of IL-4, TNF-α, and GM-CSF cannot induce neutrophils to express the high concentrations of OPG observed with neutrophils from patients with RA, other factors are involved in inducing OPG. The effect of IL-4 on neutrophil expression of OPG, however, could be associated with the anti-apoptotic function of IL-4 through OPG inhibition of TRAIL (tumor necrosis factor-related apoptosis-inducing ligand) produced by human neutrophils [35,36]. A synergism between IL-4 and TNF-α has been demonstrated for the increased production of IL-1 receptor antagonist in human neutrophils [37]. The exact mechanism (or mechanisms) underlying the synergism that stimulates the neutrophil expression of OPG remains to be elucidated and could be independent of or complementary to the NF-κB pathway that is simultaneously activated by IL-4 and by TNF-α [38,39]. Moreover, IL-4 not only activates human blood neutrophils but also is a maturation factor for precursors to become neutrophils [40] and could drive a subpopulation of neutrophils and some of their precursors present in blood to express OPG. The high concentrations of OPG measured in SF from patients with RA (see Results section) could be related to the capacity of neutrophils, which are present in large numbers, to release OPG (Figure 2c). On the other hand, given that RANK-L was not released by inflammatory neutrophils (Figure 2a), the low amounts of RANK-L measured in the same SF (see Results section) could originate only from lining fibroblast-like synoviocytes [41].

The findings that inflammatory neutrophils spontaneously express RANK (Figures 1 and 2d) and that healthy blood neutrophils express RANK only after stimulation raise the possibility that neutrophils are involved in bone remodeling. However, compared with the cytokine combination present in SM, the SFs from patients with RA are more efficient at activating neutrophils to express RANK, indicating that factors other than GM-CSF+IL-4+TNF-α are implicated in inducing RANK expression. The production of RANK protein by inflammatory neutrophils could be related to a pathophysiological role. The presence of a functional RANK protein at the cell surface of neutrophils pretreated by SFs from patients with RA, as demonstrated by RANK-L activation of the NF-κB pathway (Figure 5), indicates that such neutrophils contribute to the local tissue response.

Our findings that inflammatory neutrophils from rheumatoid SF expressed RANK at the mRNA and protein levels further confirm the plasticity of neutrophils during inflammation. Similar results were obtained with neutrophils from SF of patients with psoriatic arthritis (PE Poubelle, unpublished observations). Neutrophils can acquire the functional phenotype of active dendritic cells [10,11]. Mature dendritic cells express RANK [15,16]. Thus, the demonstration that inflammatory neutrophils express RANK could be related, in part, to their capacity of acquiring the functional phenotype of active dendritic cells, as reported in RA or Wegener granulomatosis [9,10,42,43]. The exact functions associated with neutrophil expression of RANK, however, remain to be elucidated. It is of note that neutrophil-neutrophil and neutrophil-T lymphocyte interactions have been described in pathophysiological situations [44]. Moreover, activated neutrophils have several characteristics of bone-resorbing cells. These characteristics include the capacity to form a ruffled border and the combined expression of α_v_β_3 _integrin and of certain enzymes (carbonic anhydrase II, vacuolar ATPase, cathepsin). This lends support to the hypothesis that neutrophils could be involved in bone remodeling.

The constant expression of TRAF6 by healthy and inflammatory neutrophils (Figures 1 and 3) suggests that this cytoplasmic adapter protein, which is required for immunity and bone homeostasis, is not a limiting factor in RANK-mediated neutrophil effector functions. The present report is the first to describe TRAF6 expression by neutrophils. These cells have been found to delay their programmed cell death induced by TNF-α through NF-κB and TRAF1 induction [45]. Investigation of neutrophil functions linked to TRAF6 will further our understanding of the role of this adapter protein in neutrophil biology.

## Conclusion

Direct evidence is provided for the differential expression of proteins of the RANK/RANK-L pathway in neutrophils in a non-inflammatory versus inflammatory context. Moreover, signaling occurs via RANK, the expression of which is induced in stimulated neutrophils. The results of the present study, therefore, suggest that neutrophils could play a dual role as immune and bone-like cells during the inflammatory process. This could occur via a direct interaction with dendritic cells or osteoclasts mediated by their membrane RANK-L. In certain inflammatory conditions, these neutrophils could be directly involved in acquired immunity or in bone remodeling through their expression of RANK, depending on the factors present simultaneously at the inflammatory site. As has been proposed for monocyte/macrophage precursor cells that can be driven to differentiate into dendritic cells or osteoclasts, the acquired changes of neutrophils reported above could represent an intermediary phenotype observed during the transdifferentiation of these cells [46].

## Abbreviations

Ab = antibody; BSA = bovine serum albumin; CM = control medium; EIA = enzyme immunometric assay; ELISA = enzyme-linked immunosorbent assay; FBS = fetal bovine serum; FITC = fluorescein isothiocyanate; GM-CSF = granulocyte-macrophage colony-stimulating factor; HBSS = Hanks' balanced salt solution; HRP = horseradish peroxidase; Ig = immunoglobulin; I-κB-α = inhibitor of kappaB-alpha; IL = interleukin; LDH = lactate dehydrogenase; MHC = major histocompatibility complex; NF-κB = nuclear factor-kappa-B; OA = osteoarthritis; OPG = osteoprotegerin; PBML = peripheral blood mononuclear leukocyte; PCR = polymerase chain reaction; PVDF = polyvinylidene difluoride; RA = rheumatoid arthritis; RANK = receptor activator of nuclear factor-kappa-B; RANK-L = ligand of receptor activator of nuclear factor-kappa-B; RF = rheumatoid factor; RT-PCR = reverse transcriptase-polymerase chain reaction; SD = standard deviation; SEM = standard error of the mean; SF = synovial fluid; SM = survival medium; TBS = tris-buffered saline; TNF = tumor necrosis factor; TRAF6 = tumor necrosis factor receptor-associated factor 6; TRANCE = tumor necrosis factor-related activation-induced cytokine.

## Competing interests

The authors declare that they have no competing interests.

## Authors' contributions

PEP conceived of the study, designed experiments, evaluated data, and wrote the manuscript. AC participated in the design of the study, performed experiments, and evaluated data. MJF participated in the design of the study and helped to draft the manuscript. KD performed experiments that involved molecular biology and evaluated data. A-AM carried out the immunoassays and evaluated data. All authors read and approved the final manuscript.
